# Visual outcomes after one-stage versus two-stage surgery for intraocular foreign body removal and open globe repair

**DOI:** 10.1038/s41598-026-48708-8

**Published:** 2026-04-29

**Authors:** Yujia Zhou, Anjay Shah, Ramya Singireddy, Navnit Mohan, Madison Kerley, Khoi Do, Abdulla Shaheen, Nicolas A. Yannuzzi, Wei Wang, Gibran S. Khurshid, Noy Ashkenazy, Jinghua Chen

**Affiliations:** 1 Department of Ophthalmology, University of Florida, Gainesville, FL USA; 2 University of Florida College of Medicine, Gainesville, FL USA; 3 Department of Ophthalmology, Wake Forest University, Wake Forest, NC USA; 4 Department of Ophthalmology, University of Texas Southwestern, Dallas, TX USA; 5 Department of Ophthalmology, University of Louisville, Louisville, KY USA; 6 Department of Ophthalmology, Bascom Palmer Eye Institute, Miami, FL USA

**Keywords:** Open globe injury, Intraocular foreign body, Pars plana vitrectomy, Eye trauma, Vision outcome, Surgery, Outcomes research

## Abstract

The choice between one-stage (removal during primary repair) and two-stage (delayed removal) surgical repair for intraocular foreign body injury is often based on injury characteristics, institutional capabilities, and surgeon expertise. This study aims to compare vision outcomes and complication incidence after one-stage (*n* = 70) and two-stage (*n* = 40) intraocular foreign body removal and surgical repair across multiple academic centers. We retrospectively reviewed 110 patients treated between 2000 and 2021 at four academic centers in the southeastern United States and stratified patients by initial vision into better than counting fingers or counting fingers and worse. Two-way repeated measures ANOVA found visual acuity improved (*p* < 0.001) after both one-stage (ΔVA = -0.69) and two-stage (ΔVA = -0.23) repairs, but one-stage repair was associated with greater vision improvement (*p* = 0.009) and there was no interaction with initial vision (*p* = 0.337). Endophthalmitis rates were not significantly different for each treatment group (12.9% vs. 10.0%, *p* = 0.655). The number of additional surgeries did not differ significantly between treatment groups (*p* = 0.153). One-stage repair is associated with better vision outcomes if feasible, but two-stage repair is sometimes necessary and not associated with more endophthalmitis.

**Supplementary Information:**

The online version contains supplementary material available at 10.1038/s41598-026-48708-8.

## Introduction

Intraocular foreign bodies (IOFBs) are implicated in 16–41% of open globe injuries (OGIs) and pose a significant challenge to the management of penetrating ocular injuries^1^. They often require a retina surgeon and have the potential to cause severe visual impairment or blindness. Most IOFBs originate from manual or industrial labor, and these injuries occur primarily among young adult males in the workplace^2–7^. Nearly 90% of IOFBs are metallic, while the minority include non-metallic objects like stone, plant matter, plastic, and glass^3,5,8^.

Management of IOFBs requires addressing the entry wound, typically through OGI repair unless the injury is small enough to self-seal^9^. If patients are stable enough for surgery, primary repair aims to stabilize the orbit, reapproximate ocular tissues, and restore globe integrity^10^. IOFB removal can occur during the primary repair as a one-stage procedure in cases where the globe is sealed and there is an adequate view allowing for pars plana vitrectomy (PPV), or when the IOFB is accessible without PPV^10–15^. Conversely, hidden or inaccessible IOFBs may require a two-stage repair, where globe and orbit restoration precede subsequent IOFB removal using PPV^16^.

Visual outcomes depend on a variety of factors including initial vision, type of IOFB, location of IOFB, severity of OGI, and time to surgical repair^7,17–20^. The Ocular Trauma Score (OTS) provides a visual acuity (VA) prognosis for OGIs such as IOFBs and uses initial VA as the most important predictor. There is evidence suggesting that early removal reduces the risk of complications such as endophthalmitis, proliferative vitreoretinopathy, and ocular toxicity^11,12,16,21–25^. However, IOFB removal may be deferred for inert materials such as glass and plastic, which have been reported to safely remain in the eye for extended periods of time^23,24^. Delaying IOFB removal may mitigate inflammation, allow time for vitreous separation and wound healing, and permit patient recovery prior to prolonged surgery^10^. Variability in surgical expertise and institutional availability of on-call retina specialists may contribute to delayed IOFB removal following primary OGI by non-retina specialists. The decision between a one-stage and two-stage repair is often individualized to the patient’s condition and IOFB characteristics, but there is not yet strong evidence for better outcomes in either treatment scheme^10^.

This multi-institutional study aims to address this knowledge gap by analyzing current practice patterns and outcomes in IOFB management across four major academic centers in the southeastern United States: the University of Florida (UF), University of Miami Bascom Palmer Eye Institute (BPEI), the University of Texas Southwestern (UTSW), and University of Louisville (U of L). By incorporating data from multiple centers, the study seeks to compare one and two-stage IOFB repair outcomes across diverse surgical settings, surgeon expertise, and patient populations. The findings may inform clinical decision-making in IOFB cases, potentially influencing staffing decisions, resource allocation, and surgical protocols in ophthalmic trauma care.

## Materials and methods

### Study design

This study is a multi-center retrospective cohort analysis of IOFB outcomes, pooling data from four major academic eye centers: UF, BPEI, UTSW, and U of L. Institutional review board at each institution granted a waiver of informed consent and a waiver of HIPAA authorization due to minimal risk and retrospective design. The research adhered to tenets of the Declaration of Helsinki. The cohort includes patients who presented to the above centers with IOFB and received surgical treatment between January 1, 2000, and December 31, 2021. Patients with < 90 days of follow-up at time of data collection were excluded due to insufficient follow-up for vision rehabilitation. One-stage protocol was defined as repair in which the IOFB was removed at the time of primary OGI repair, even if the patient required subsequent rehabilitative surgeries. Two-stage protocol was defined as any repair in which IOFB removal was performed separately after the primary open globe repair. The choice of protocol often depended on the severity and complexity of injuries, with one-stage repair being more feasible in eyes that were more amenable to immediate IOFB removal.

### Variables and outcomes

Patient age and sex were the only demographic variables collected for the study. Other characteristics for the injured eye such as VA, intraocular pressure (IOP), mechanism of injury, follow-up timeline, and initial exam findings were recorded. Primary outcomes of interest included final best-corrected VA (BCVA) or pinhole VA when BCVA is not available. Secondary outcomes included number of subsequent surgeries and IOFB complications including: corneal ulcer, band keratopathy, choroidal hemorrhage, macular scarring, epiretinal membrane (ERM), endophthalmitis, toxic retinopathy, phthisis, sympathetic ophthalmia (SO), enucleation, and evisceration. Exam findings and sequelae were only recorded if they resulted from the same IOFB injury in the same eye, except for a diagnosis of SO, which was recorded in the contralateral eye or bilateral eyes. Injury mechanisms were assigned at the best medical discretion of each site’s record keeper.

VA was recorded in Logarithm of the minimum angle of resolution (LogMAR) scale^26^. The better-vision stratum was defined as any VA better than counting fingers (LogMAR < 1.9), and worse-vision defined as any VA counting fingers (CF) or worse (LogMAR ≥ 1.9). The threshold of CF was chosen to provide a clinically useful distinction when patients are “off-chart”^27^. This approach reflects its clinical relevance in emergency settings where patients often cannot perform standard visual acuity tests.

IOP was tested using standardized contact tonometry devices or Goldmann applanation. For Goldmann applanation, a slit-lamp mounted tonometer was applied to the eye after topical anesthesia with 0.5% proparacaine and fluorescein sodium staining, and measurements were taken in a seated position. For standardized tonometry, a rebound or indentation tonometer was applied to the eye after 0.5% proparacaine anesthesia, and the average of at least 3 measurements was recorded. IOP was recorded as zero if the globe was widely open or obviously deflated (i.e. equalized to atmospheric pressure or absence of intraocular space) preventing use of quantitative methods.

### Statistical analysis

JASP statistical software version 0.19.0 with α = 0.05 was used for all statistical analyses (The JASP Team, Amsterdam, The Netherlands)^28^. The Chi-square test of independence was used to compare cohort characteristics, exam findings, and secondary outcomes compared by site and treatment scheme. For highly skewed variables such as time interval, medians were reported in addition to means and standard deviations (SDs) which do not accurately represent the skewed distribution. VA distribution shape was tested with Shapiro-Wilk and kurtosis. Initial VA and time intervals were compared by Mann-Whitney *U*-test due to non-normal distributions. Age and IOP were compared by Student’s *t*-test.

Primary statistical approach used multiple repeated-measures analysis of variance (ANOVA) to test for within-subject effects (i.e., time point), between-subject effects (i.e., vision stratum and treatment scheme), and other univariate interactions affecting final VA^29^.Sphericity for ANOVA was not necessary with two repeated measures, Holm-Bonferroni post-hoc tests were conducted for significant comparisons, and homoscedasticity was confirmed with Levene’s test^30^. Due to differences in injury severity between groups, a severity-stratified study design was necessary to reduce allocation bias. Initial VA thus served as a proxy for injury severity and along with the repeated measures design helps to isolate effect of repair modality across varied injury severities^31^.

To evaluate the factors influencing VA and to validate ANOVA findings, we conducted a Generalized Linear Model (GLM) in the Gaussian family with identity link, using a minimum cell size of 6. The model utilized post-operative VA as the dependent variable with preoperative VA and staging of surgery as mandatory independent variables. Factors not associated with final VA were pruned in reverse fashion, and model parsimony was optimized using the Bayesian Information Criterion (BIC). Residuals, multicollinearity, interactions were examined. Macular scarring was explored with multivariable logistic regression optimized for BIC in a similar fashion. The repeated measures ANOVA approach is widely used and interpreted in ophthalmology when assumptions are met, as reported below in results. This cohort did not need GLM for paired-eye or complex time series data, and thus ANOVA remained as the primary statistical approach^29^.

## Results

### Cohort characteristics

**Fig. 1:**
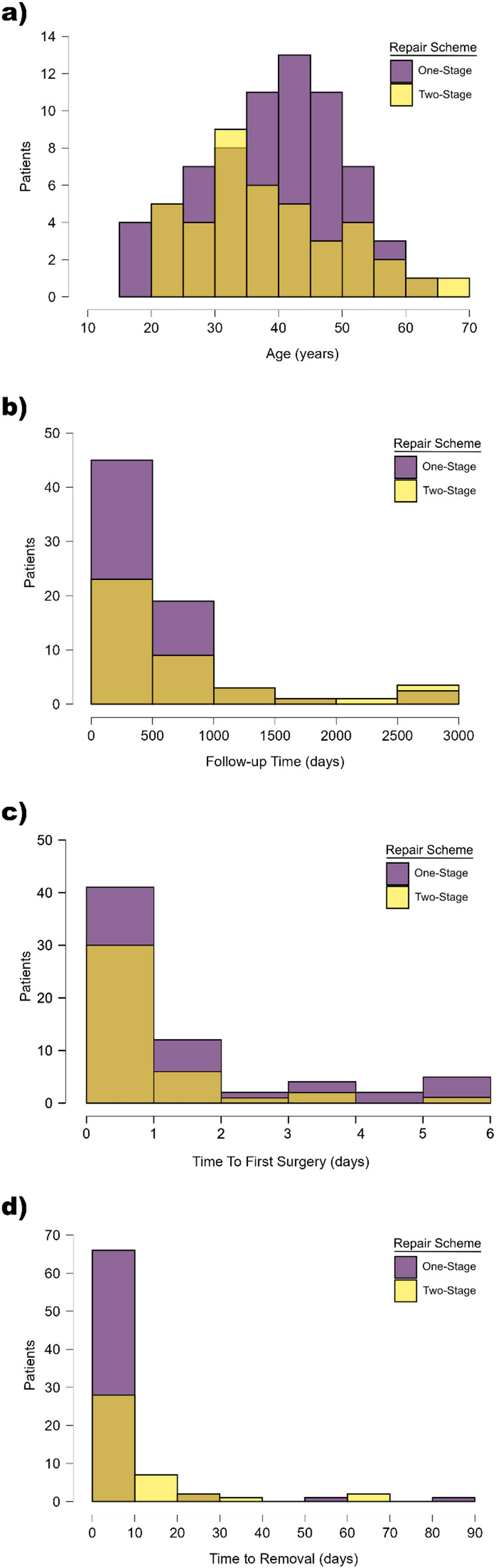
Overlapping distributions for one-stage (purple) and two-stage (yellow) repair scheme are shown for **(a)** age in years, **(b)** duration of follow-up after injury, **(c)** time from injury to first surgery, and **(d)** time from injury to IOFB removal. Patients with follow-up less than 90 days were excluded.

**Fig. 2:**
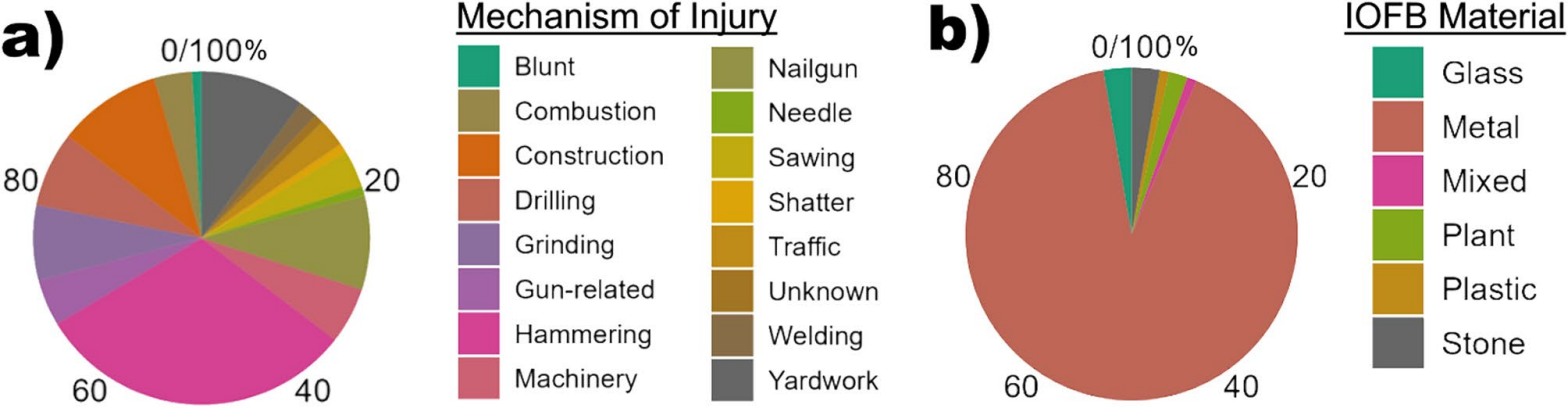
Details of the intraocular foreign body injury are divided into pie chart categories regarding **(a)** mechanism of injury recorded by each site, and **(b)** material of the foreign body removed from the globe.

Amongst the four participating centers, 112 patients were injured by an IOFB, and 2 patients were lost to follow-up. For the remaining 110 patients, 34 (30.9%) came from UF, 32 (29.1%) came from BPEI, 32 (29.1%) came from UTSW, and 12 (10.9%) came from U of L. Most patients were male (106/110, 96.4%), and most repairs were performed in one-stage (70/110, 63.6%) versus two-stages (40/110, 36.4%). Mean age at time of presentation was 38.5 (SD 11.4) years, and the median length of follow-up was 370.5 days (mean 609 days), ranging from the inclusion threshold of 90 days to a maximum of 4124 days (Fig. 1a and b), with no difference between groups (*p* = 0.939, *p* = 0.116). The median time to surgery was 1 day (range 1–90, mean 3) as shown in Fig. 1c indicating more than half received initial surgery within the same day, and the median time to IOFB removal was 3 days (range 1–90, mean 7) with distribution shown in Fig. 1d. For the 40/110 (36.4%) patients receiving two-stage repair and IOFB removal, median time between surgeries was 7 (range 0–67, mean 10.7) days. The patient who underwent second surgery 67 days later had corneal opacity that prevented vitrectomy. Mechanisms of injury involved manual labor in 94/110 cases (85.5%), and IOFB material was metallic in 100/110 (90.9%) of injured patients (Fig. 2a and b).

### Pre-surgical characteristics

**Fig. 3:**
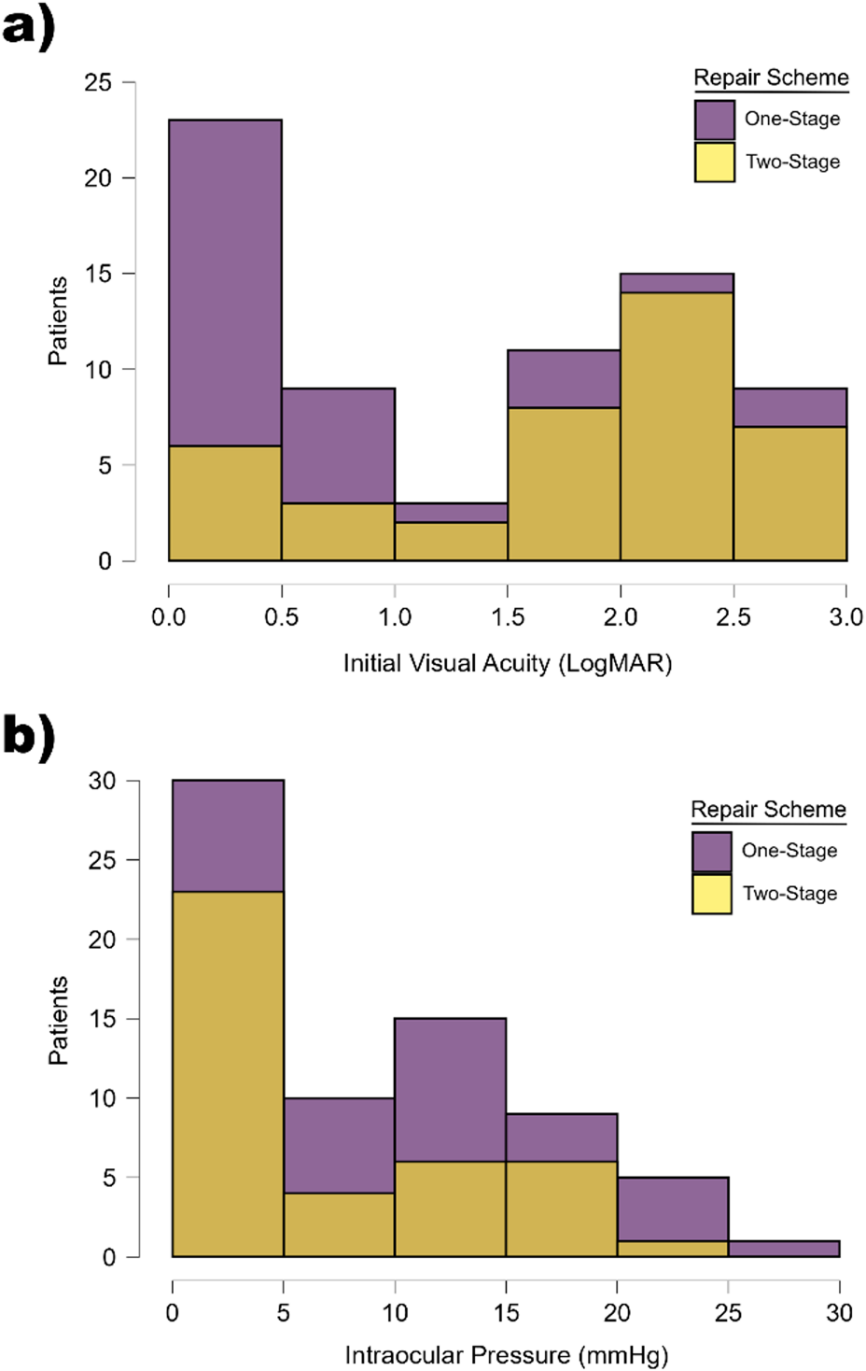
Overlapping distributions for one-stage (purple) and two-stage (yellow) repair scheme are shown for **(a)** initial visual acuity in LogMAR and **(b)** intraocular pressure in mmHg, including patients with zero pressure. A higher LogMAR indicates worse vision and lower LogMAR indicates better vision.

Initial LogMAR visual acuity (Fig. 3a) was bimodally distributed (*p* < 0.001, kurtosis = −1.50) with a mean of 1.51 (SD 0.99). Mean IOP (Fig. 3b) was 7.4 (SD 8.0) mmHg, with 51/110 (46.4%) patients having measured or presumed IOP of zero. There were no significant differences in initial VA (*p* = 0.228) and IOP (*p* = 0.313) between sites, but initial VA was significantly worse (Fig. 3a) for patients who later underwent two-stage repair (1.81 vs. 1.34, *p* = 0.019). VA outcomes were then stratified by initial VA, with 48/110 (43.6%) patients in the better-vision stratum and 62/110 (56.4%) patients in the worse-vision stratum. Patients who underwent two-stage repair were more likely to have had scleral lacerations (47.5% vs. 27.1%, *p* = 0.031), hyphema (40.0% vs. 12.9%, *p* = 0.001), or vitreous hemorrhage (65.0% vs. 42.9%, *p* = 0.025), while other injury features were not associated with any treatment scheme (Table 1). Patients in the worse-vision stratum had almost had significantly higher rates of scleral laceration (26/62 or 41.9% vs. 12/48 or 25.0%, *p* = 0.064) and lens injury (49/62 or 79.0% vs. 30/48 or 62.5%, *p* = 0.056) compared to those in the better-vision stratum.

**Table 1:** Initial ocular examination findings.

Exam finding	Incidence (% within column)			Significance (χ²)
	Overall 110 eyes	One-stage 70 eyes	Two-stage 40 eyes	
Corneal Laceration	74 (67.3)	47 (67.1)	27 (67.5)	*p* = 0.969
Scleral Laceration	38 (34.5)	19 (27.1)	19 (47.5)	*p* = 0.031*
Lens Injury	79 (71.8)	51 (72.9)	28 (70.0)	*p* = 0.749
Iris Injury	58 (52.7)	38 (54.3)	20 (50.0)	*p* = 0.665
Retinal Injury	67 (60.9)	45 (64.3)	22 (55.0)	*p* = 0.337
Hyphema	25 (22.7)	9 (12.9)	16 (40.0)	*p* = 0.001*
Vitreous Hemorrhage	56 (50.9)	30 (42.9)	26 (65.0)	*p* = 0.025*
Macular Subretinal Hemorrhage	5 (4.5)	2 (2.9)	3 (7.5)	*p* = 0.261
Choroidal Hemorrhage	10 (9.1)	6 (8.6)	4 (10.0)	*p* = 0.802

*Significant comparisons at α = 0.05.

**Fig. 4:**
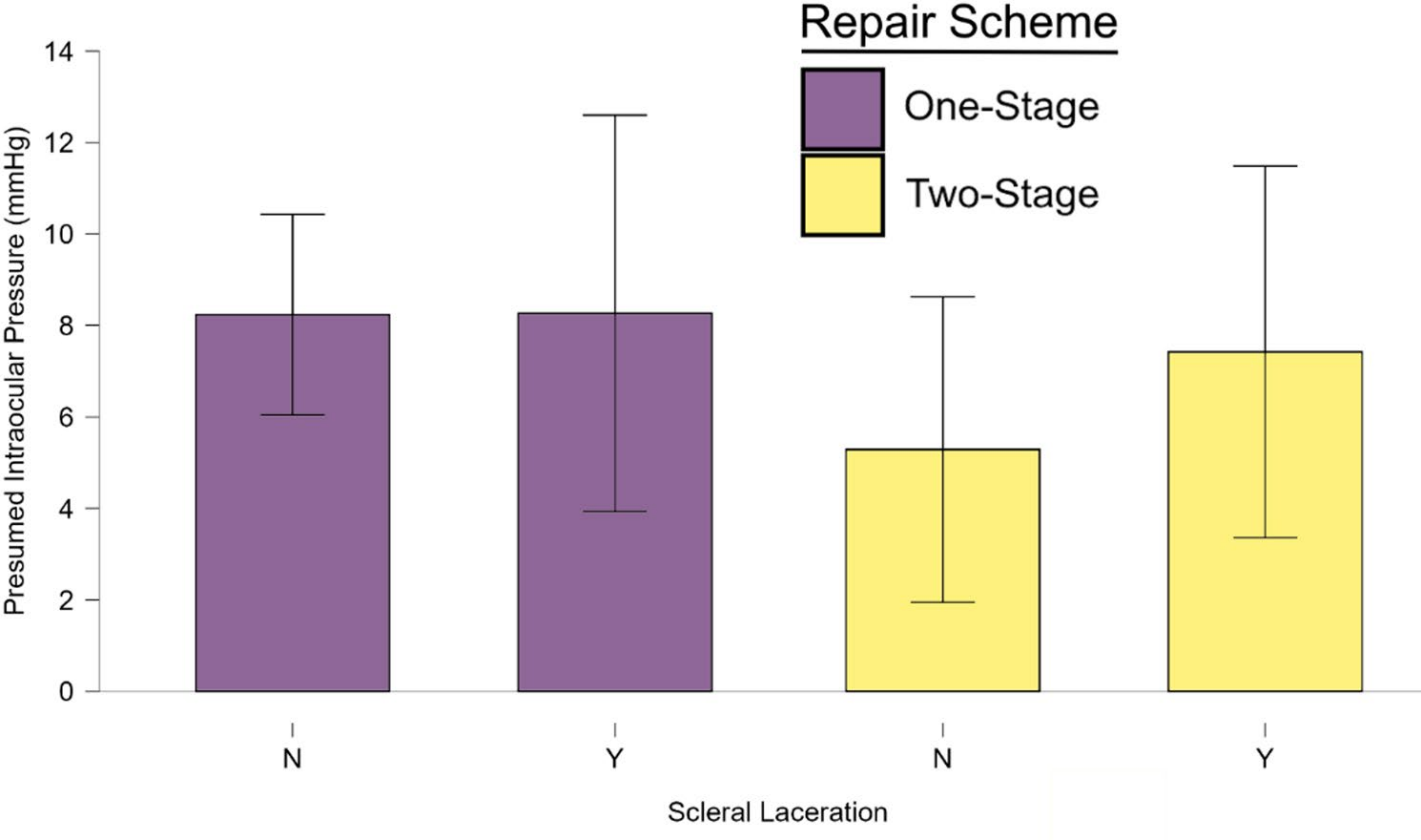
Bar charts of intraocular pressure separated by repair scheme and presence of scleral laceration. Error bars represent 95% confidence interval.

Differences between groups were compared to examine any effects on vision outcome or treatment effect. ANOVA found that IOP (including hypotonous eyes) was not significantly associated with scleral laceration (*p* = 0.518), staged repair (*p* = 0.259), and there was no cross-interaction associated with intraocular pressure (*p* = 0.529, Fig. 4), indicating that neither group had more wide-open globes due to scleral lacerations. Multiple repeated measures ANOVA also found that the effect of treatment scheme on vision outcome does not interact with scleral laceration (*p* = 0.177), hyphema (*p* = 0.200), or vitreous hemorrhage (*p* = 0.423).

### Post-surgical outcomes

**Fig. 5:**
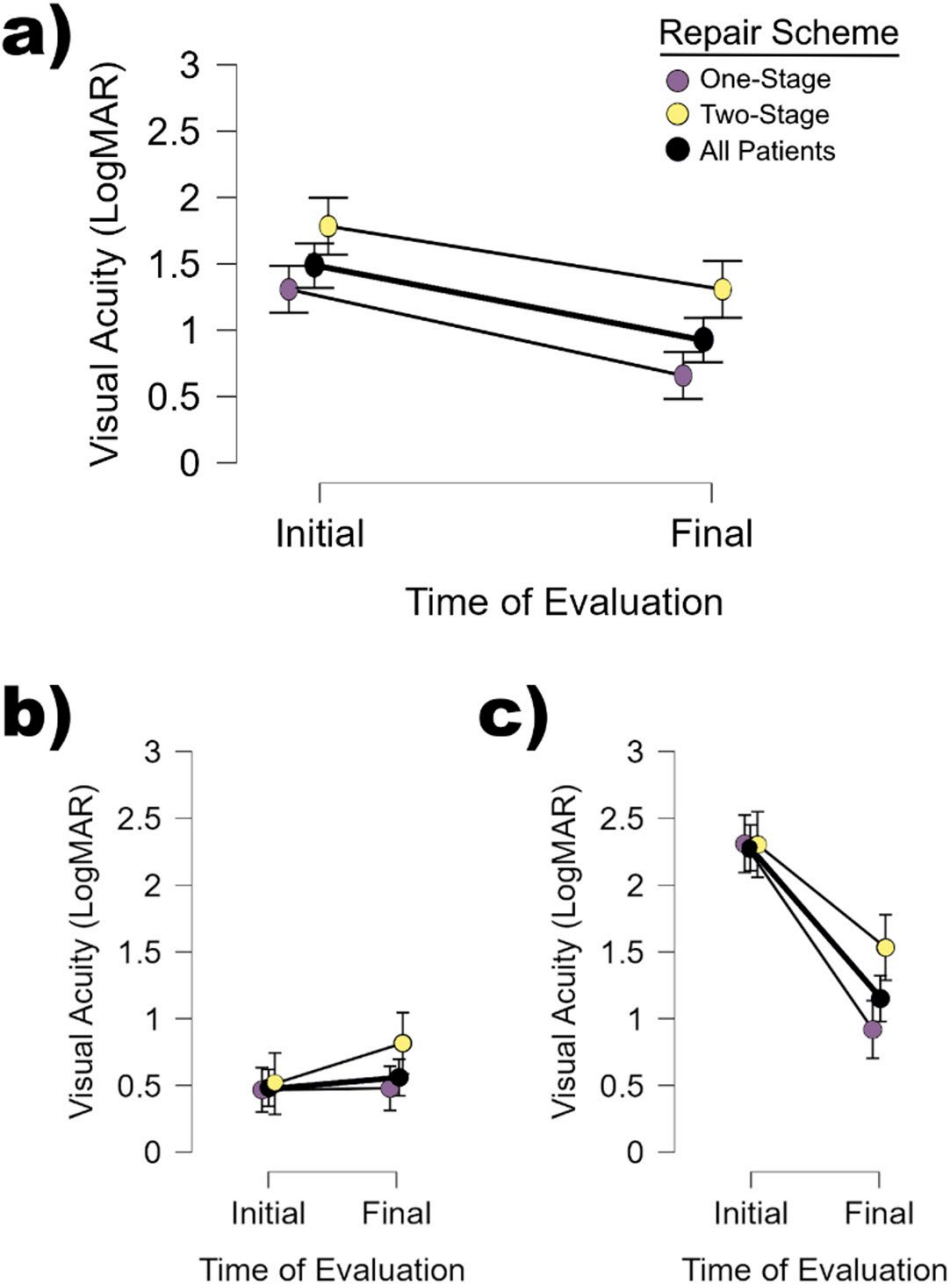
Mean visual acuities and 95% confidence intervals **(a)** at initial and final time points for each repair scheme, then separated into groups of eyes with initial vision **(b)** better than counting fingers or **(c)** counting fingers and worse. Small visual offsets were applied to each point to avoid overlapping points. An uptrending LogMAR indicates worsening vision and downtrending LogMAR indicates improving vision.

The multiple repeated measures ANOVA found that VA significantly improved after treatment (*p* < 0.001), one-stage vs. two-stage treatment scheme (*p* = 0.027), and vision stratum (*p* < 0.001). Levene’s test for initial VA within each comparison was nonsignificant (*p* = 0.183) and normality was confirmed by Q-Q plot (Supplement 1). Pairwise VA change from initial to final follow-up was significantly affected by treatment scheme (*p =* 0.009) and vision stratum (*p* < 0.001), but there were no significant crossover interactions between treatment scheme and vision stratum (*p* = 0.337). Vision significantly improved from initial to final timepoints (ΔVA = −0.59, *p* < 0.001) for both treatment groups (Fig. 5a). Vision improvement was − 0.69 (*p* < 0.001) in the one-stage group and − 0.23 (*p* = 0.011) in the two-stage group. Patients in the better-vision stratum (Fig. 5b) generally did not have significant vision gains following repair (ΔVA = 0.16, *p =* 0.514) while patients in the worse-vision stratum (Fig. 5c) generally had substantial improvements in vision (ΔVA = −1.10, *p* < 0.001).

The parsimonious reduced GLM significantly predicted post-operative outcomes (X^2^(2) = 23.65, *p* < 0.001) and demonstrated superior fit (BIC_1_ = 279.75 vs. BIC_0_ =302.75) compared to the base model (Supplement 2). Initial VA was significantly associated with final VA (*b* = 0.358, *p* < 0.001), and a two-stage surgical approach was associated with worse final VA (*b* = 0.480, *p* = 0.004). Like the primary statistical model, there was no significant interaction between initial VA and surgical approach. VIF values of 1.06 confirmed the absence of multicollinearity and addition of any other variable or interaction increased BIC without decreasing deviance.

**Fig. 6:**
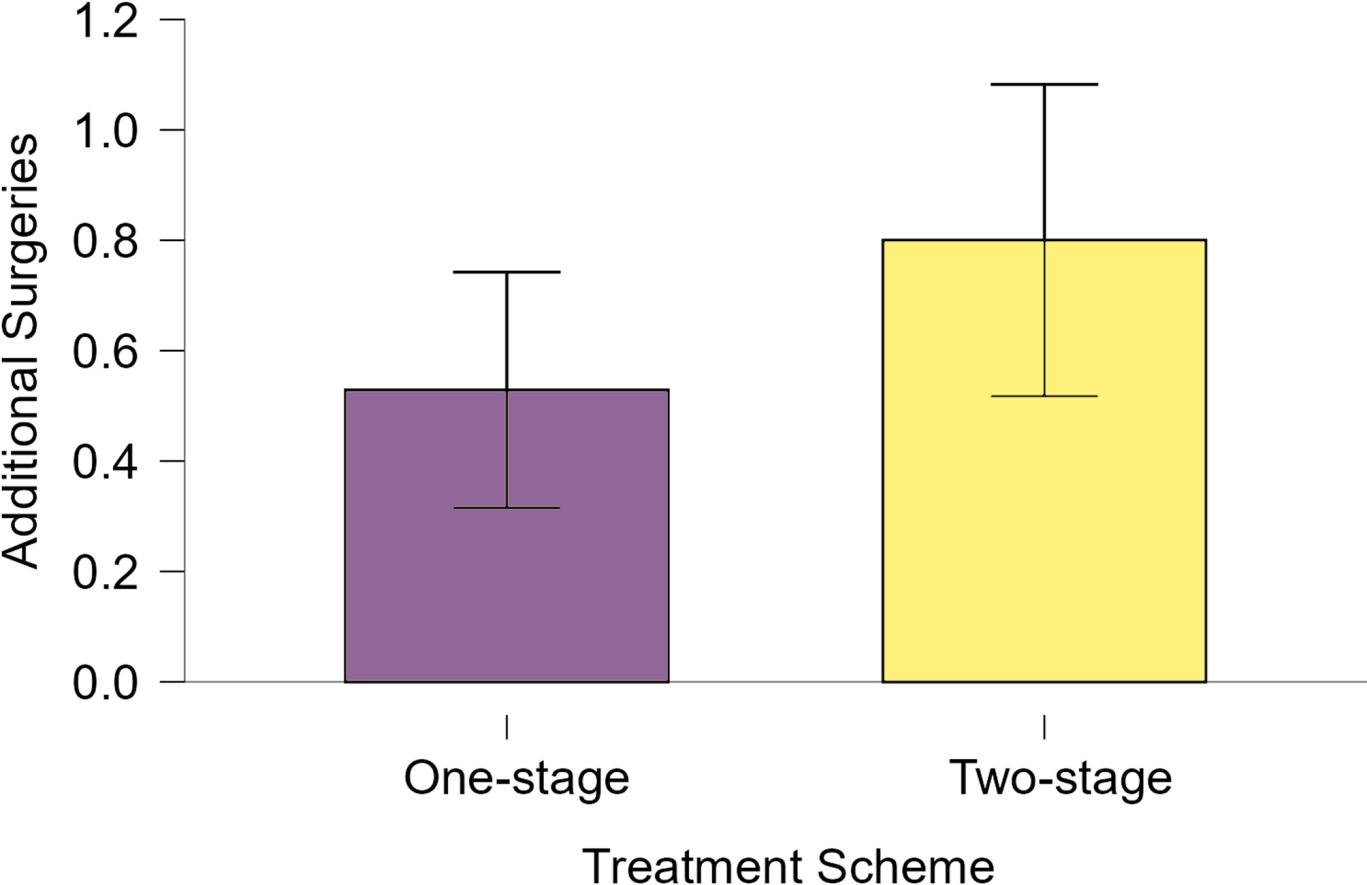
Bar chart showing the mean number of additional surgeries needed after each treatment scheme, with 95% confidence interval shown. Two-stage repairs appear to require more surgeries afterwards, although the difference is not statistically significant.

**Fig. 7:**
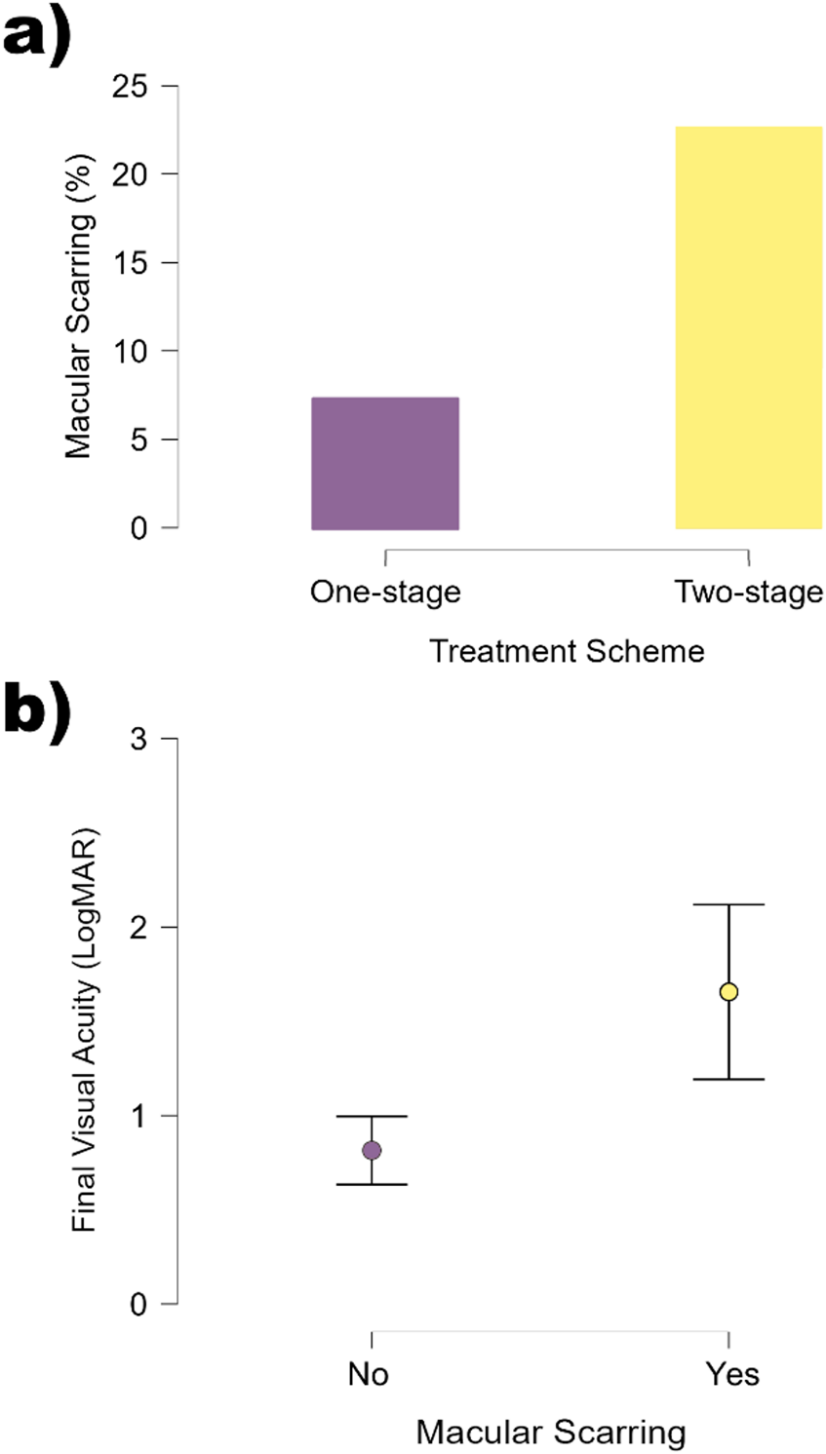
Analysis of macular scarring as a clinical outcome. **(a)** Macular scarring is significantly more common after two-stage repair (22.5%) than after one-stage repair (7.1%). **(b)** Patients with macular scarring have significantly worse final visual acuity. A higher LogMAR indicates worse vision and lower LogMAR indicates better vision.

**Fig. 8:**
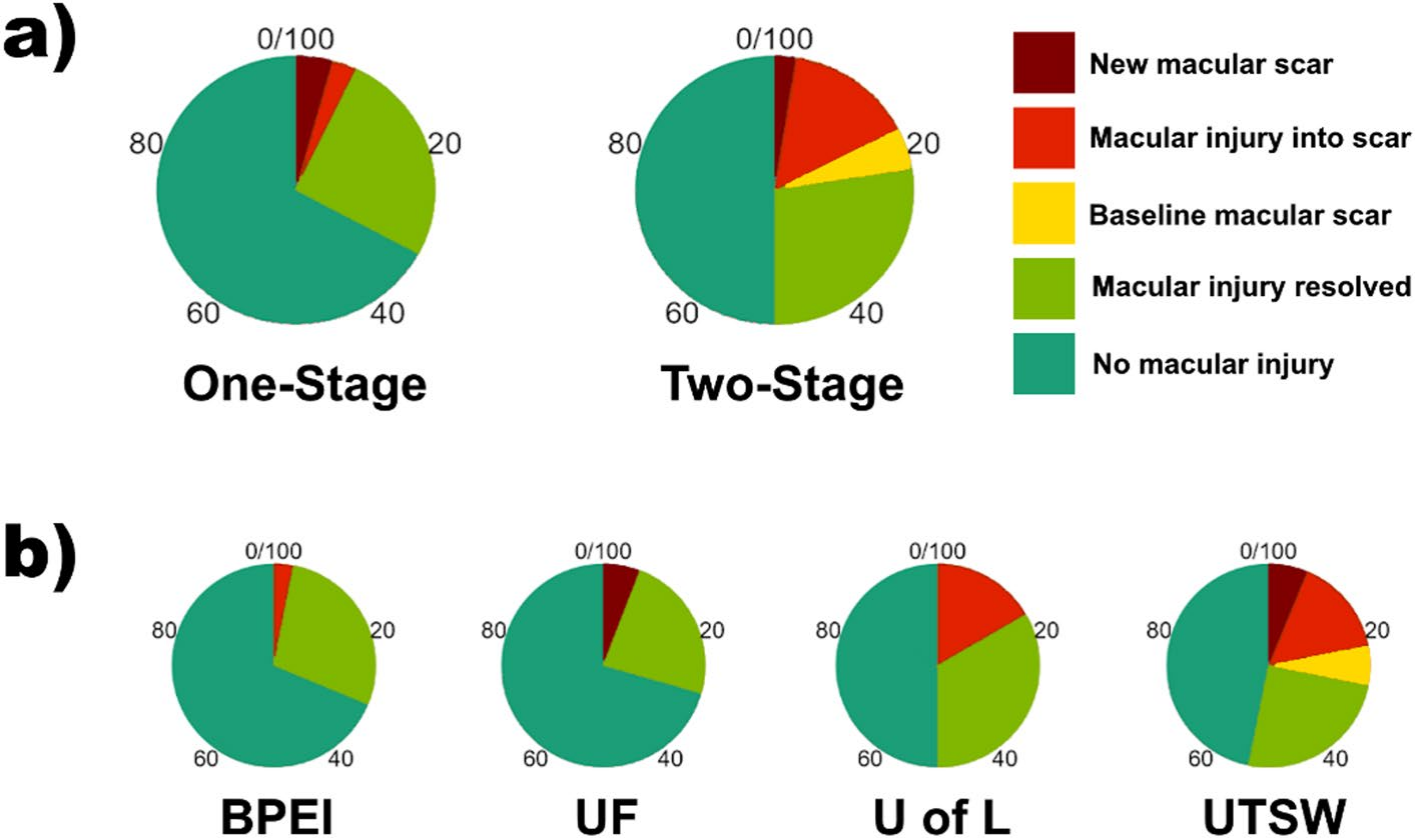
Pie charts displaying the incidence of macular scarring divided by clinical history for **(a)** one-stage vs. two-stage repairs and **(b)** for each participating institution. Patients who presented with a known clinical history or view of an uninjured macula and developed macular scarring after IOFB removal were considered to have a new macular scar (dark red), while those who did not develop a scar are considered to have no macular injury (teal green). Patients found to have evidence of macular injury by direct view or other imaging were considered to have a macular injury that either resolved without scar (lime green) or developed into a scar (bright red). Baseline macular scars were identified on history or prior view (yellow). Pie charts are scaled with numbers from 0–100%.

The number of additional surgeries (Fig. 6) after one-stage and two-stage repair was not significantly different (0.52 vs. 0.80, *p* = 0.153). Sequelae are reported in Table 2, and rates of sequelae were not significantly different between groups except for macular scarring (Fig. 7a), which was more common after two-stage repair (22.5% vs. 7.1%, *p* = 0.020). Post-hoc analysis (Fig. 7b) of VA revealed that poor final VA was significantly associated with macular scarring (ΔVA = −0.76, *p* = 0.001). Incidence of macular scarring was significantly related to site (*p* = 0.011) as well (Fig. 8b). Similarly, logistic regression (Supplement 3) showed macular scarring remains associated with two-stage repair (*p* = 0.040) when accounting for all injury characteristics and may be associated with iris injuries (*p* = 0.031) as well.

**Table 2:** Secondary outcomes.

Secondary 0utcomes	Incidence (% within column)			Significance (χ²)
	Overall 110 eyes	One-stage 70 eyes	Two-stage 40 eyes	
Corneal Ulcer	1 (0.9)	0 (0)	1 (2.5)	*p* = 0.184
Band Keratopathy	1 (0.9)	0 (0)	1 (2.5)	*p* = 0.184
Strabismus	1 (0.9)	1 (1.4)	0 (0.0)	*p* = 0.448
Choroidal Hemorrhage	9 (8.2)	6 (8.6)	3 (7.5)	*p* = 0.844
Macular Scarring	14 (12.7)	5 (7.1)	9 (22.5)	*p* = 0.020*
Epiretinal Membrane	26 (23.6)	14 (20)	12 (30.0)	*p* = 0.235
Endophthalmitis	13 (11.8)	9 (12.9)	4 (10.0)	*p* = 0.655
Toxic Retinopathy	0 (0.0)	0 (0.0)	0 (0.0)	-
Sympathetic Ophthalmia	1 (0.9)	1 (1.4)	0 (0.0)	*p* = 0.448
Phthisis	4 (3.6)	2 (2.9)	2 (5.0)	*p* = 0.564
Evisceration/Enucleation	1 (0.9)	0 (0)	1 (2.5)	*p* = 0.184

*Significant comparisons at α = 0.05.

### Comparing sites

**Fig. 9:**
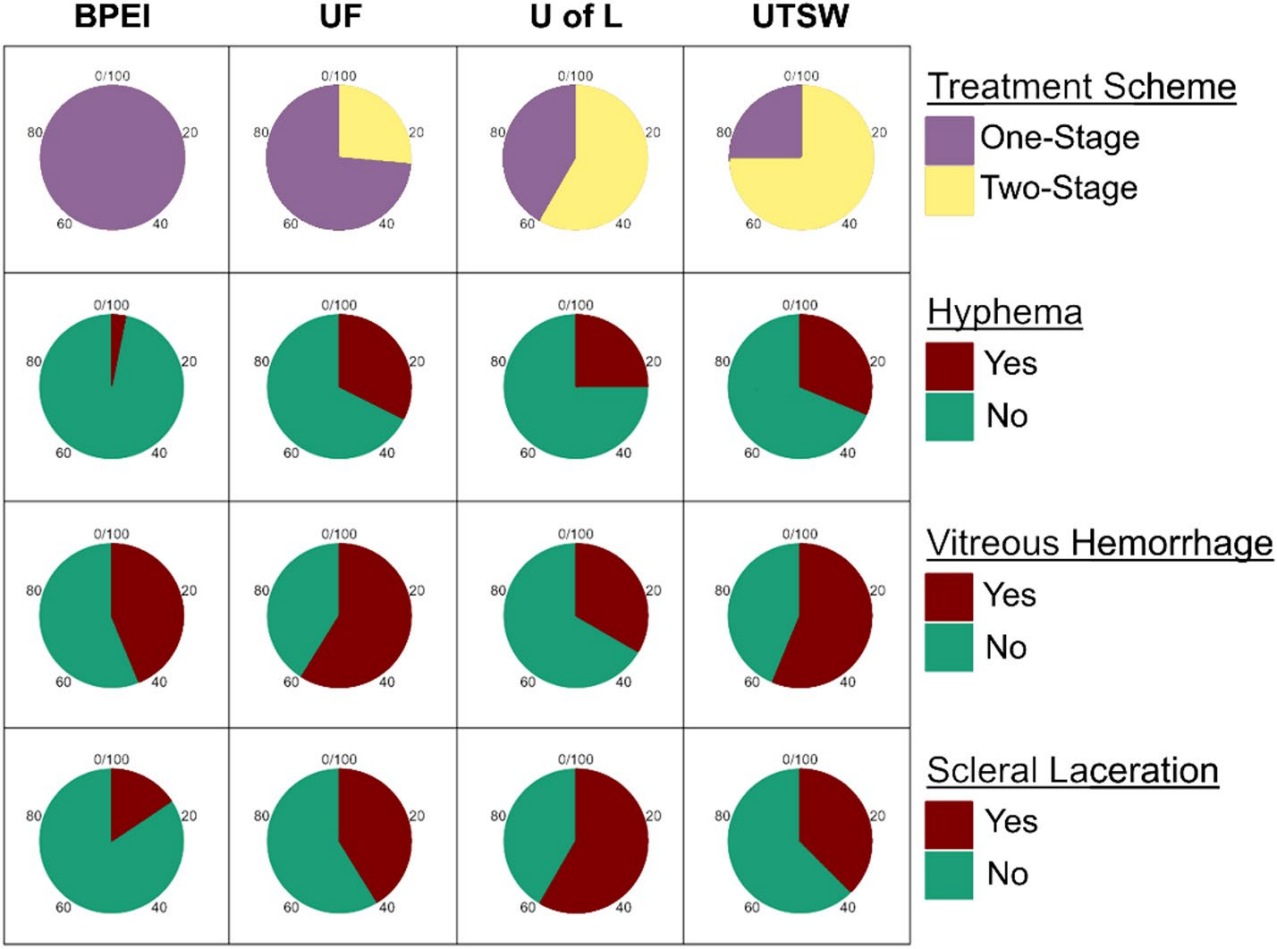
Treatment schemes used and the associated injuries encountered at each site are displayed in a matrix of pie charts. Only injuries associated with a treatment scheme were included. One-stage repair was always performed at BPEI, while other sites had a mix of one and two-stage repairs. At sites where two-stage repair were more common, hyphema was more common, vitreous hemorrhage had similar incidences, and scleral lacerations were more common. Pie charts are scaled with numbers from 0–100%.

Figure 9 illustrates the characteristics of treatment schemes and associated injuries at each site, selecting for the associated injuries that were significantly related to treatment scheme. Proportions of staged surgeries differed at each site (*p* < 0.001). At BPEI, where there was no departmental policy to favor one-stage repair, all (100%) IOFBs received one-stage repair. Significantly fewer injuries at BPEI were found to have hyphema (3.1% vs. 22.7%, *p* = 0.017) compared to the overall cohort. Corneal laceration rates did not significantly differ between sites. BPEI had a significantly lower rate of scleral lacerations while U of L had a higher rate than other institutions (15.6 vs. 58.3%, *p* = 0.031). There were no significant differences in follow-up length, age, gender, mechanism, IOFB material, time to surgery, or time between surgeries (*p* > 0.050) across treatment sites.

## Discussion

The decision between one-stage and two-stage repair represents an important dilemma in the management of IOFB injuries. While ophthalmologists generally agree that early repair reduces the risk of endophthalmitis, PVR, and ocular toxicity, there is limited and conflicting evidence that one-stage repair leads to improved VA^10,32^.A one-stage repair does avoid repeated exposure to anesthesia and is theorized to have similar benefits of earlier repair, but there may be advantages of two-stage repair even when one-stage repair is feasible, such as an improved view for PPV and reducing intraocular inflammation. In the absence of clear indications for either treatment scheme, decisions are often individualized for the injured patient^10^.

Except for a 5-year study of 1421 patients across 15 centers in China by Zhang et al. (2011), existing studies are conducted at individual eye centers. This study was therefore conducted to compare outcomes of one-stage and two-stage IOFB treatment schemes across four academic eye centers in the southeast United States, with the aim to generate insights more generalizable across different practice patterns.

### Demographics and injury characteristics

In this cohort, IOFBs were mostly work-related injuries and predominantly affected males of working age in their 2nd to 5th decade of life (Fig. 1a), consistent with previous reports^5,7,8,12,33,34^.

Specific mechanisms of injury varied (Fig. 2a), but most involved a hard projectile with high velocity generated by hammering, nailgun, or gunshots. Mechanisms of injury may reflect the at-risk behaviors that vary by region. For example, Zhang et al. (2011)reported a much higher rate of explosion IOFBs (27.8%) in China where mining and firework use is more common^7^. Likewise, Colyer et al. (2007)reported almost exclusively weapon-related IOFBs (98.7%) because the cohort included United States military veterans on active duty^12^. Metallic IOFBs were most prevalent in our study (100/110, 90.9%), aligning with previous reports^1,3–5,7,16,33,34^.

Initial VA appeared to have a bimodal distribution in this cohort (Fig. 3a), separated into subset of patients with good vision and a subset with poor vision. Zhang et al. (2011)also reported a bimodally distributed initial VA in a large sample size of 1421 patients^7^. Similar bimodally distributed VA appears in other vision-limiting diseases where patients either have well-preserved vision or severe vision impairment^35,36^. Comparing initial VA with other studies is difficult due to a variety of reporting methods, but the proportion of patients with vision better than 1.00 (20/200) ranges from 20% to 50%, similar to the 37% in this cohort^1,3,5,12,16,34,37^. Our initial exam findings in Table 1showed similar rates of lens injury, corneal laceration, and retinal injury. However, we observed notable differences in certain features. Scleral laceration, vitreous hemorrhage, and hyphema were more common in our cohort compared to several reports^3,5,6,34^. These variations likely reflect differences in study populations and injury mechanisms.

### Choice of surgical repair

Trauma is a high-variance setting where not all factors are easily quantified or categorized, and there is no well-accepted decision pathway. In areas without retina coverage, two-stage repair may be necessary as non-retina surgeons must leave the IOFB for later removal by a retina surgeon with pars plana vitrectomy, even if the primary surgery could seal the globe and address all other injuries. It may be hard to do one-stage surgery repeatedly in a smaller institution if multiple traumas occur in a short time span, as the operation depends on specialized nursing and surgical technician staffing needed to coordinate with complex and limited vitreoretinal equipment. All 4 study centers are academic centers with retina coverage 24 h a day, 7 days a week (i.e. all the time) but surgeon availability and willingness is not the only factor. This study includes an example of one patient who underwent staged repair 67 days later due to temporary corneal opacity, demonstrating additional requirements in unique situations. For example, an alternate retina surgeon may have found the initial corneal transparency satisfactory or could have done a joint temporary keratoprosthesis case if a cornea surgeon was also willing. In other cases, joint or intentionally staged repairs are preferred, for example if the patient has life-threatening injuries which take priority. In general, one-stage repair with pars plana vitrectomy is possible when the view is sufficient to identify the IOFB, the globe is mostly sealed, and when the surgeon decides doing so would not do undue harm.

While the surgeon factor may not be quantifiable, the data does show patients selected for two-stage repair had worse injuries as defined by lower initial VA (Fig. 5a) and higher rates of scleral laceration, hyphema, and vitreous hemorrhage (Table 1). Initial vision may not be perfectly indicative of injury severity, but it remains one of the most impactful and accurate predictors of vision prognosis in OGIs and IOFBs, as well as anecdotally used among surgeons^4,38,39^. Poor initial VA may be correlated with other features of injury severity.

The anatomical complexity of an injury may also influence decisions regarding treatment scheme, suggested by higher rates of scleral laceration, vitreous hemorrhage, and hyphema in our two-stage group. Some retina surgeons prefer to delay PPV after initial OGI repair due to a poor view or widely unsealed globe that prevents PPV^10^. In this retrospective study, the size of laceration was not recorded in most cases, which can be another factor affecting surgical approach in unexpected ways. A wide-open scleral laceration is thought to be more difficult to seal and could require a two-stage approach, or it may actually be more suitable for one-stage repair if the IOFB is exposed and can be manually removed without retina surgery^40^. The data does not show that “sealability” of a globe confounded treatment scheme, as IOP was not associated with repair scheme, scleral laceration, or any cross interaction (Fig. 4). Ultimately, none of these secondary features were associated with outcomes and inclusion in the primary statistical model was unnecessary.

### Primary outcome: visual acuity

Observing the trends before and after surgery, VA improved for both one-stage and two-stage repair groups (Fig. 5a). The overall vision improved from a mean of 1.51 to 0.92 in LogMAR, roughly the difference between 20/600 and 20/160 in Snellen equivalents. Colyer et al. (2007) reports a similar significant vision gain from a mean of 1.36 to 0.75, while Bourke et al. (2021)reports a vision gain without significance testing from 0.79 to 0.58^3,12^. In our cohort, 78.2% of patients presented with vision worse than 1.30 (20/400), which decreased to 69/110 (62.7%) post-surgery. When stratified by initial VA, however, VA changes diverged. Patients in the better-vision stratum had overall decreases in VA (Fig. 5b), while patients in the worse-vision stratum overall had significant improvements in VA (Fig. 5c). This suggests that patients with worse vision receive more visual benefit after surgery.

Poorer initial visual acuity was confounded with two-stage repair (Fig. 3a) and poorer vision outcome, so a stratified study design with two-way repeated measures ANOVA was necessary^41^. Vision change was significantly different between treatment groups, with the one-stage group having a greater LogMAR improvement of −0.69 compared to the two-stage group with − 0.23 (Fig. 5a). Constraining initial vision by vision strata in Fig. 5b and c reveals that when controlling for initial vision, the consistently worse outcomes from two-stage repair in both vision strata become more apparent. These results show that one-stage repair is associated with superior outcomes independently of initial vision.

The choice for one-stage or two-stage repair could depend on the feasibility of surgery, as this study has shown that scleral laceration and poor view due to intraocular bleeding are associated with choice of two-stage treatment scheme. Therefore, efforts were required to ensure factors related to injury severity were not confounded with the effect of treatment group on outcomes. Patients with scleral lacerations, hyphema, or vitreous hemorrhage were more likely to undergo two-stage repair. These features may make vitrectomy more technically challenging but are not associated with vision prognosis in the OTS, so may not be suitable for measuring “severity” as a research construct^39^. As expected, none of these factors were associated with vision outcome or interacted with the effect of repair scheme chosen.

### Secondary outcomes: sequelae

Each surgery increases the risk of ocular sequelae, anesthesia complications, impact on quality of life, and cost of rehabilitation. This study shows there is no significant difference between additional surgeries after one or two-staged repair. Most common sequelae were not significantly different between treatments, except macular scarring was more common in the two-stage group than in the one-stage group (Fig. 8a and Supplement 3b). In this study two patients were known to have macular scar prior to injury, but other macular scars were likely caused by trauma as most patients had no ocular history^8^. Macular scarring was also associated with worse visual outcomes (Fig. 8b). A more sensitive explorative analysis using logistic regression (Supplement 3) also shows that iris injuries may also be significantly related to macular scarring, implicating the uveal inflammatory pathway after iris-involving injuries. Considering that scleral lacerations and hyphema were more common for both two-stage surgeries (Table 1) and some of the institutions with higher rates of macular scarring (Fig. 9), macular scarring could reflect a certain type of injury rather than choice of two-stage repair.

Our endophthalmitis rate was 13/110 (11.8%), with no significant difference between single-stage and two-stage repair groups (12.9% vs. 10.0%, *p* = 0.655). This rate is higher than some previous reports, such as the 2.6% reported by Chang et al. (2021), but lower than the 16.76% reported by Ratanapakorn et al. (2021)^5,34^. The variability in endophthalmitis rates across studies may reflect differences in antibiotic prophylaxis protocols, time to treatment, and patient population. This study was also not designed to assess the duration of IOFB retainment within the globe, as one-stage repairs were performed up to several weeks after initial injury, and some two-stage repairs were performed sequentially on the same day of injury. Study groups do have similar time from injury to surgery, so timing of surgery is not expected to confound results. The nonmetallic IOFBs were also too infrequent to conduct meaningful analyses regarding endophthalmitis risk based on IOFB material.

We did not clinically observe any cases of toxic retinopathy despite many metallic IOFBs, which has been reported in some IOFB studies^10,42^. It may be possible that subclinical toxicity was undetected, as electroretinogram results were not included in this study. SO was rare with only a single case in the one-stage group, and given that an estimated 80% of cases of SO present within 3 months of the inciting trauma, this study is expected to capture most potential cases^43^. There were few cases of phthisis (3.6%), with 2 patients in the one-stage group and 2 patients in the two-stage group. There were no enucleations, but there was one case of secondary evisceration. The rate of eye loss was at 4.5% in our cohort, comparable with prior reports ranging from 0.3–17%^3,5,11^. Incidence was too low to make any comparison between groups for these rare outcomes.

### Limitations and study interpretation

Our study has several limitations. First, the study design is not suited to analyze outcomes with low or delayed incidence (e.g. sympathetic ophthalmia) and factors such as repair timing. Low-incidence outcomes would require a case-control design or a significantly larger multi-center cohort. Late complications such as sympathetic ophthalmia may present decades after injury and may not appear in this cohort. Patients are followed up for 90 days as indicated by the Medicare and Medicaid global period, and early exclusion prior to vision rehabilitation at 90 days is not a limitation except for two patients who were lost to follow-up. Surgical timing is a separate question from surgical approach, and has been well described in literature that any primary repair should be done as soon as feasible^42^. While this study is not designed to analyze surgical timing, we did analyze surgical timing as an independent variable and found no interaction with surgical approach or outcome.

Second, this was not a randomized controlled trial, and surgeons may measure “severity” subjectively at an individual basis, contributing to allocation bias^44^. While we did not calculate OTS due to retrospective data limitations, VA is the primary weighted component of the OTS and was used in the stratified statistical model to minimize allocation bias. The OTS also is not proven to represent ease of repair or suitability for two-stage repair. Instead, the injury features mentioned in Fig. 9are more directly related to allocation, determined by individual surgeons. Generalizing the cohort with a multicenter design including many retina surgeons across multiple centers helps mitigate this limitation, similarly reported in Zhang et al. because there are no controlled or prospective studies on this topic^7^. Prospective studies are not guaranteed to eliminate allocation bias, so a sufficiently large and generalized cohort would be needed for more reliable results^31,45^.

Third, there is variation between study centers. Certain centers had more one-stage repairs (Fig. 9, *p* < 0.001), such as BPEI, which only had one stage repairs in their included cases. We also observed significant differences between sites in terms of initial exam features (Fig. 9, *p* < 0.05). From one perspective, multicenter studies are meant to capture this variability and generalize findings across institutions. From another perspective, minimizing or accounting for these factors is necessary to compare and pool site data. There were no protocols that necessitated any treatment scheme, and all sites ensured 24/7 retina surgeon coverage. Nevertheless, one-stage IOFB removal and open globe repair may still be challenging for smaller trauma centers. Results show no differences in surgery timing, initial vision, or IOP between sites, indicating surgeon response to injury severity was not skewed by any individual site. The characteristics of those presenting injuries at each institution are more likely responsible for site variation, as hyphema and scleral laceration are more common at centers where two-stage repair were more common (Fig. 9). Vitreous hemorrhage incidence was not significantly different between sites, although it was more often associated with a two-stage repair. These injury features may represent covariation with the groups but did not affect VA outcome or treatment effect and are not confounds.

## Conclusion

This study analyzes retrospective data from the four academic centers (UF, BPEI, UTSW, and U of L) to examine practice patterns and outcomes of intraocular foreign body removal and repair. This is the first multicenter study of intraocular foreign body removal in the United States and demonstrates that two-stage repair is not inferior to one-stage repair in many aspects: patients in both groups had categorically improved vision after surgeries, similar percentage of postop patients developed choroidal hemorrhage, epiretinal membrane, endophthalmitis, toxic retinopathy, evisceration, and enucleation. However, this analysis does demonstrate that a one-stage surgical approach for IOFB repair is associated with greater vision outcomes at a follow up of > 90 days, so ophthalmologists should not leave an IOFB which is feasible to remove during primary repair. Sampled practice patterns show that the decision to perform a two-stage repair is associated with poor initial vision, scleral laceration, hyphema, and vitreous hemorrhage which may make one-stage repair unfeasible.

Given the challenges of conducting randomized controlled trials in ocular trauma, there is a need for large-scale multi-center or prospective studies to further elucidate optimal management strategies for IOFB injuries. Additionally, institution-specific quality and safety studies could inform staffing decisions, particularly regarding the availability of surgeons and staff needed to facilitate one-stage IOFB repair.

While surgical management is crucial, prevention remains paramount. This cohort demonstrates that even less severe injuries with good initial vision are at risk of vision loss. Ophthalmologists should proactively screen for high-risk behaviors and emphasize the importance of appropriate ocular protective equipment in relevant occupational and recreational settings, particularly for young or working-age male patients.

## Supplementary Information

Below is the link to the electronic supplementary material.

Supplementary Material 1

Supplementary Material 2

Supplementary Material 3

## Data Availability

This research has been conducted using the Resource from University of Florida, University of Texas Southwestern, University of Louisville and Bascom Palmer Eye Institute. The datasets generated and analyzed in the current study are available from the corresponding author and these institutions on reasonable request.

## Abbreviations

IOFB: Intraocular foreign body
OGI: Open globe injury
PPV: Pars plana vitrectomy
VA: Visual acuity
IOP: Intraocular pressure
ERM: Epiretinal membrane
SO: Sympathetic ophthalmia
LogMAR: Logarithm of the minimum angle of resolution
SD: Standard deviation
CF: Counting fingers
ANOVA: Analysis of variance
