# Supplementary material for: Visual outcomes after one-stage versus two-stage surgery for intraocular foreign body removal and open globe repair
Source: Sci Rep. 2026 Apr 29;16:19894. doi: 10.1038/s41598-026-48708-8 (PMC13316033; doi:10.1038/s41598-026-48708-8)
Supplement: Supplementary file 1 — Supplementary Material 1 [file 41598_2026_48708_MOESM1_ESM.pdf]

Results

Supplement 1. RM two-way ANOVA for comparing VA outcomes and surgical approach stratified by initial VA strata

Within Subjects Effects

Cases	Sum of Squares	df	Mean Square	F	p
RM Factor 1	9.347	1	9.347	29.504	< .001
RM Factor 1 ✻ Staged	2.273	1	2.273	7.174	0.009
RM Factor 1 ✻ VIS_Stage	16.785	1	16.785	52.983	< .001
RM Factor 1 ✻ Staged ✻ VIS_Stage	0.295	1	0.295	0.930	0.337
Residuals	33.582	106	0.317		

Note. Type III Sum of Squares

Between Subjects Effects

Cases	Sum of Squares	df	Mean Square	F	p
Staged	2.702	1	2.702	5.030	0.027
VIS_Stage	62.801	1	62.801	116.936	< .001
Staged ✻ VIS_Stage	0.136	1	0.136	0.254	0.615
Residuals	56.928	106	0.537		

Note. Type III Sum of Squares

Descriptives

Descriptives

RM Factor 1	VIS_Stage	Staged	N	Mean	SD	SE	Coefficient of variation
Initial	H	N	37	0.466	0.469	0.077	1.006
		Y	11	0.513	0.466	0.141	0.909
	L	N	33	2.309	0.317	0.055	0.137
		Y	29	2.303	0.313	0.058	0.136
Final	H	N	37	0.477	0.549	0.090	1.151
		Y	11	0.816	0.766	0.231	0.939
	L	N	33	0.918	0.983	0.171	1.071
		Y	29	1.532	0.979	0.182	0.639

Assumption Checks

Test for Equality of Variances (Levene's)

	F	df1	df2	p
VA1	1.646	3	106	0.183
VA2	5.831	3	106	< .001

Test of Sphericity

Cannot perform sphericity tests because there are only two levels of the RM factor, or because the SSP matrix is singular.

Q-Q Plot
