# Supplementary material for: Visual outcomes after one-stage versus two-stage surgery for intraocular foreign body removal and open globe repair
Source: Sci Rep. 2026 Apr 29;16:19894. doi: 10.1038/s41598-026-48708-8 (PMC13316033; doi:10.1038/s41598-026-48708-8)
Supplement: Supplementary file 2 — Supplementary Material 2 [file 41598_2026_48708_MOESM2_ESM.pdf]

Results

Supplement 2a. Inclusive Generalized Linear Model for predicting final VA with overfitting as shown on residual plot

Model Summary - VA2

Model	Deviance	AIC	BIC	df	X²	p
H_0	92.703	297.348	302.749	109		
H_1	52.242	266.261	314.870	93	40.461	< .001

Model Fit

	Statistic	df	p
Deviance	52.242	93	1.000

Coefficients

	Estimate	Standard Error	t	p
(Intercept)	0.671	0.299	2.243	0.027
TTS	0.002	0.008	0.200	0.842
VA1	0.356	0.081	4.393	< .001
IOP1	-0.010	0.010	-1.001	0.319
StagedY	0.417	0.216	1.929	0.057
LensY	0.172	0.193	0.888	0.377
IrisY	0.084	0.199	0.420	0.676
RetY	-0.013	0.158	-0.084	0.933
B ACY	0.312	0.195	1.599	0.113
B MacY	0.132	0.377	0.349	0.728
B VitY	-0.189	0.167	-1.129	0.262
B ChorY	0.723	0.264	2.739	0.007
KY	-0.507	0.250	-2.023	0.046
ScY	0.012	0.229	0.050	0.960
SiteUF	-0.337	0.199	-1.894	0.059
SiteUL	-0.675	0.305	-2.213	0.029
SiteUT	-0.003	0.262	-0.011	0.991

Diagnostics

Residuals vs. Fitted Plots

Standardized deviance residuals vs. fitted values

Multicollinearity Diagnostics

	VIF
TTS	1.244
VA1	1.247
IOP1	1.283
Staged	2.117
Lens	1.480
Iris	1.935
Ret	1.161
B AC	1.310
B Mac	1.210
B Vit	1.365
B Chor	1.127

K	2.703
Sc	2.326
Site	2.852

Supplement 2b. Intermediate Generalized Linear Model with all factors of interest that meet cell limits

Model Summary - VA2

Model	Deviance	AIC	BIC	df	X^2	p
H_0	92.703	297.348	302.749	109		
H_1	67.033	273.683	295.287	103	25.670	< .001

Model Fit

	Statistic	df	p
Deviance	67.033	103	0.998

Coefficients

	Estimate	Standard Error	t	p
(Intercept)	0.387	0.242	1.601	0.112
VA1	0.354	0.082	4.321	< .001
StagedY	0.445	0.176	2.520	0.013
ScY	-0.051	0.235	-0.217	0.829
B ACY	0.215	0.202	1.064	0.290
B VitY	-0.045	0.165	-0.276	0.783
KY	-0.251	0.228	-1.098	0.275

Diagnostics

Residuals vs. Fitted Plots

Standardized deviance residuals vs. fitted values

Multicollinearity Diagnostics

	VIF
VA1	1.098
Staged	1.217
Sc	2.111
B AC	1.211
B Vit	1.144
K	1.940

Supplement 2c. Parsimonious Generalized Linear Model containing only VA and surgical approach, assuming no interaction

Model Summary - VA2

Model	Deviance	AIC	BIC	df	X^2	p

H_0	92.703	297.348	302.749	109		
H_1	69.050	268.945	279.747	107	23.653	< .001

Model Fit

	Statistic	df	p
Deviance	69.050	107	0.998

Coefficients

	Estimate	Standard Error	t	p
(Intercept)	0.208	0.144	1.447	0.151
VA1	0.358	0.080	4.471	< .001
StagedY	0.480	0.164	2.930	0.004

Diagnostics

Residuals vs. Fitted Plots

Standardized deviance residuals vs. fitted values

Multicollinearity Diagnostics

	VIF
VA1	1.057
Staged	1.057

Supplement 2d. Interaction General Linear Model of initial VA and surgical approach revealing no interaction and a poorer fit compared to the parsimonious model

Model Summary - VA2

Model	Deviance	AIC	BIC	df	X^2	p
H_0	92.703	297.348	302.749	109		
H_1	67.737	268.833	282.335	106	24.966	< .001

Model Fit

	Statistic	df	p
Deviance	67.737	106	0.999

Coefficients

	Estimate	Standard Error	t	p
(Intercept)	0.308	0.159	1.936	0.056
VA1	0.282	0.095	2.964	0.004
StagedY	0.066	0.332	0.197	0.844
VA1 ✻ StagedY	0.248	0.173	1.434	0.155

Diagnostics

Residuals vs. Fitted Plots

Standardized deviance residuals vs. fitted values

Multicollinearity Diagnostics

	VIF
VA1	1.515
Staged	4.382
VA1:Staged	5.358
