# Supplementary material for: Visual outcomes after one-stage versus two-stage surgery for intraocular foreign body removal and open globe repair
Source: Sci Rep. 2026 Apr 29;16:19894. doi: 10.1038/s41598-026-48708-8 (PMC13316033; doi:10.1038/s41598-026-48708-8)
Supplement: Supplementary file 3 — Supplementary Material 3 [file 41598_2026_48708_MOESM3_ESM.pdf]

Results

Supplement 3. Logistic Regression for macular scar predictors

Model Summary - Mac

Model	Deviance	AIC	BIC	df	ΔX²	p	McFadden R²	Nagelkerke R²	Tjur R²	Cox & Snell R²
M_0	62.149	94.149	137.357	94			0.000	0.000	0.258	0.000
M_1	67.114	93.114	128.221	97	3.400	0.334	-0.051	-0.067	0.191	-0.030
M_2	67.114	91.114	123.520	98	0.000	0.998	-0.051	-0.067	0.191	-0.030
M_3	67.126	89.126	118.831	99	0.012	0.914	-0.051	-0.067	0.191	-0.031
M_4	67.152	87.152	114.157	100	0.026	0.871	-0.051	-0.067	0.191	-0.031
M_5	67.194	85.194	109.499	101	0.042	0.837	-0.052	-0.068	0.192	-0.031
M_6	67.588	83.588	105.192	102	0.393	0.531	-0.057	-0.075	0.187	-0.035
M_7	67.914	81.914	100.818	103	0.326	0.568	-0.062	-0.081	0.179	-0.037
M_8	68.213	80.213	96.416	104	0.299	0.585	-0.066	-0.087	0.174	-0.040
M_9	68.765	78.765	92.268	105	0.553	0.457	-0.073	-0.097	0.173	-0.045

Coefficients

					Wald Test		
Model	Parameter	Estimate	Robust Standard Error	z	Wald Statistic	df	p
M_0	(Intercept)	-5.766	1.637	-3.523	10.350	1	< .001
	VA1	0.664	0.424	1.565	2.479	1	0.118
	IOP1	-0.015	0.056	-0.271	0.101	1	0.786
	K (Y)	0.506	1.099	0.460	0.160	1	0.645
	Lens (Y)	-0.870	1.107	-0.786	0.812	1	0.432
	B AC (Y)	-0.387	0.747	-0.518	0.193	1	0.604
	B Vit (Y)	-0.072	1.006	-0.071	0.006	1	0.943
	B Chor (Y)	0.334	1.028	0.325	0.078	1	0.745
	Sc (Y)	0.307	0.917	0.335	0.094	1	0.738
	Iris (Y)	1.751	1.160	1.510	2.472	1	0.131
	B Mac (Y)	1.496	0.995	1.504	1.037	1	0.133
	Staged (Y)	0.010	0.637	0.016	1.341×10^{-4}	1	0.987
	Ret (Y)	0.254	0.746	0.341	0.120	1	0.733
	Site (UF)	1.103	1.354	0.815	0.643	1	0.415
	Site (UL)	2.516	1.473	1.707	2.630	1	0.088
	Site (UT)	2.545	1.220	2.086	3.418	1	0.037
M_2	(Intercept)	-4.874	1.306	-3.731	11.904	1	< .001
	VA1	0.523	0.377	1.386	1.834	1	0.166
	IOP1	0.005	0.053	0.095	0.012	1	0.924
	K (Y)	0.723	1.013	0.714	0.352	1	0.475
	Lens (Y)	-0.564	0.929	-0.607	0.391	1	0.544
	B AC (Y)	-0.467	0.713	-0.655	0.303	1	0.512
	B Vit (Y)	-0.189	0.786	-0.241	0.054	1	0.810
	B Chor (Y)	0.157	0.945	0.166	0.022	1	0.868
	Sc (Y)	0.611	0.832	0.735	0.401	1	0.462
	Iris (Y)	1.781	0.908	1.961	3.361	1	0.050
	B Mac (Y)	1.772	1.101	1.610	1.716	1	0.107
	Staged (Y)	1.280	0.637	2.008	3.229	1	0.045
M_3	(Intercept)	-4.818	1.236	-3.898	13.460	1	< .001
	VA1	0.526	0.388	1.357	1.860	1	0.175
	K (Y)	0.728	1.013	0.719	0.357	1	0.472
	Lens (Y)	-0.570	0.962	-0.593	0.403	1	0.553
	B AC (Y)	-0.466	0.712	-0.655	0.304	1	0.512
	B Vit (Y)	-0.197	0.803	-0.245	0.059	1	0.807
	B Chor (Y)	0.172	0.915	0.188	0.027	1	0.851
	Sc (Y)	0.603	0.825	0.731	0.392	1	0.465
	Iris (Y)	1.753	0.746	2.350	3.512	1	0.019
	B Mac (Y)	1.800	0.978	1.840	1.831	1	0.066
	Staged (Y)	1.273	0.668	1.907	3.227	1	0.056
M_4	(Intercept)	-4.841	1.228	-3.942	13.686	1	< .001
	VA1	0.524	0.392	1.339	1.845	1	0.181
	K (Y)	0.771	0.980	0.786	0.414	1	0.432
	Lens (Y)	-0.570	0.958	-0.595	0.406	1	0.552
	B AC (Y)	-0.501	0.726	-0.690	0.370	1	0.490
	B Vit (Y)	-0.158	0.799	-0.198	0.042	1	0.843
	Sc (Y)	0.628	0.812	0.774	0.432	1	0.439
	Iris (Y)	1.743	0.740	2.356	3.506	1	0.018
	B Mac (Y)	1.791	0.974	1.839	1.804	1	0.066
	Staged (Y)	1.271	0.666	1.907	3.209	1	0.056
M_5	(Intercept)	-4.855	1.215	-3.998	13.852	1	< .001
	VA1	0.506	0.334	1.515	1.808	1	0.130
	K (Y)	0.810	0.945	0.858	0.472	1	0.391
	Lens (Y)	-0.567	0.964	-0.588	0.399	1	0.557
	B AC (Y)	0.549	0.716	0.766	0.483	1	0.444

	B AC (Y)	[illegible]	[illegible]	[illegible]	[illegible]	1	[illegible]
	Sc (Y)	0.624	0.793	0.787	0.431	1	0.431
	Iris (Y)	1.670	0.736	2.269	3.822	1	0.023
	B Mac (Y)	1.795	0.973	1.845	1.801	1	0.065
	Staged (Y)	1.249	0.675	1.850	3.185	1	0.064
M_6	(Intercept)	-5.090	1.195	-4.258	15.978	1	< .001
	VA1	0.466	0.318	1.463	1.612	1	0.143
	K (Y)	0.642	0.846	0.759	0.322	1	0.448
	B AC (Y)	-0.578	0.701	-0.824	0.542	1	0.410
	Sc (Y)	0.728	0.771	0.944	0.619	1	0.345
	Iris (Y)	1.612	0.695	2.319	3.780	1	0.020
	B Mac (Y)	1.912	0.975	1.961	2.102	1	0.050
	Staged (Y)	1.273	0.653	1.950	3.380	1	0.051
M_7	(Intercept)	-4.704	0.992	-4.740	19.775	1	< .001
	VA1	0.495	0.312	1.587	1.887	1	0.112
	B AC (Y)	-0.594	0.691	-0.861	0.585	1	0.389
	Sc (Y)	0.417	0.717	0.581	0.303	1	0.561
	Iris (Y)	1.798	0.741	2.425	5.393	1	0.015
	B Mac (Y)	1.789	0.906	1.974	1.983	1	0.048
	Staged (Y)	1.338	0.655	2.041	3.838	1	0.041
M_8	(Intercept)	-4.533	0.957	-4.736	20.688	1	< .001
	VA1	0.509	0.313	1.628	2.013	1	0.104
	B AC (Y)	-0.561	0.689	-0.815	0.523	1	0.415
	Iris (Y)	1.672	0.755	2.214	5.177	1	0.027
	B Mac (Y)	2.030	0.841	2.413	2.907	1	0.016
	Staged (Y)	1.367	0.645	2.119	4.039	1	0.034
M_9	(Intercept)	-4.539	0.969	-4.686	20.792	1	< .001
	VA1	0.487	0.315	1.545	1.890	1	0.122
	Iris (Y)	1.651	0.775	2.130	5.035	1	0.033
	B Mac (Y)	1.818	0.880	2.067	2.541	1	0.039
	Staged (Y)	1.299	0.633	2.053	4.218	1	0.040

Note. Mac level 'Y' coded as class 1.

Multicollinearity Diagnostics

	Tolerance	VIF
VA1	0.976	1.025
Iris	0.948	1.055
B Mac	0.966	1.036
Staged	0.960	1.042
